# Effect of chilling acclimation on germination and seedlings response to cold in different seed coat colored wheat (*Triticum aestivum* L.)

**DOI:** 10.1186/s12870-021-03036-z

**Published:** 2021-06-02

**Authors:** Paulina Calderon Flores, Jin Seok Yoon, Dae Yeon Kim, Yong Weon Seo

**Affiliations:** 1 Department of Plant Biotechnology, Korea University, Seoul, 02841 Korea; 2 Department of Biotechnology, Korea University, Seoul, 02841 Korea

**Keywords:** Cold tolerance, Flavonoids, Low temperature, Purple wheat, ROS

## Abstract

**Background:**

Flavonoids can protect plants against extreme temperatures and ROS due to their antioxidant activities. We found that deep-purple seed coat color was controlled by two gene interaction (12:3:1) from the cross between yellow and deep-purple seed coat colored inbreds. F_2:3_ seeds were grouped in 3 by seed coat color and germinated under chilling (4 °C) and non-acclimated conditions (18 °C) for a week, followed by normal conditions (18 °C) for three weeks and a subsequent chilling stress (4 °C) induction. We analyzed mean daily germination in each group. Additionally, to study the acclimation in relationship to the different seed coat colors on the germination ability and seedling performances under the cold temperatures, we measured the chlorophyll content, ROS scavenging activity, and expression levels of genes involved in ROS scavenging, flavonoid biosynthetic pathway, and cold response in seedlings.

**Results:**

The results of seed color segregation between yellow and deep purple suggested a two-gene model. In the germination study, normal environmental conditions induced the germination of yellow-seed, while under chilling conditions, the germination ratio of deep purple-seed was higher than that of yellow-colored seeds. We also found that the darker seed coat colors were highly responsive to cold acclimation based on the ROS scavenging enzymes activity and gene expression of ROS scavenging enzymes, flavonoid biosynthetic pathway and cold responsive genes.

**Conclusions:**

We suggest that deep purple colored seed might be in a state of innate pre-acquired stress response state under normal conditions to counteract stresses in a more effective way. Whereas, after the acclimation, another stress should enhance the cold genes expression response, which might result in a more efficient chilling stress response in deep purple seed seedlings.

Low temperature has a large impact on the yield of crops. Thus, understanding the benefit of seed coat color response to chilling stress and the identification of limiting factors are useful for developing breeding strategies in order to improve the yield of wheat under chilling stress.

**Supplementary Information:**

The online version contains supplementary material available at 10.1186/s12870-021-03036-z.

## Background

The increasing population and climate change are challenges for future agriculture production. To keep up with demands of the rising population, it has been estimated that agricultural production would have to double by 2050 [1]. Cold tolerant species are able to acclimate to low temperatures by an osmotic adjustment such as increasing sugars, organic acids, and amino acids [2, 3]. However, sudden changes in temperature can harm plants primarily from severe dehydration [4, 5]. Low temperatures, also called chilling temperatures, are low (0–15 °C) but not freezing temperatures (below 0 °C) [6]. Previous studies have reported that chilling temperatures can inhibit phloem export [7–10], decrease carbon fixation [7], interrupt the circadian rhythm by regulating the transcription of photosynthetic genes [11], and degrade damaged reaction PSII centers [12].

It is known that winter wheat (*Triticum aestivum* L.) requires vernalization, exposure of the plant to low temperatures as a mean of control of flower formation [13, 14]. It has been reported that the best vernalization response of winter wheat is between 7 and 8 °C as it shows slower responses at higher or lower temperatures [15]. Moreover, there is evidence suggesting that devernalization can occur when seedlings are placed under warm environmental conditions after a cold treatment [13].

Winter wheat can tolerate temperatures up to -20 °C through cold acclimation [16]. Cold-tolerant crops (winter wheat and winter rye, for example) can acquire maximum freezing tolerance after a prolonged seedling growth and development under low temperatures (0–5 °C) [7]. Since plants are exposed to low and subzero temperatures in the field during fall and winter [7], there is a need for plants to acclimate to sudden changes in temperature known to perturb their metabolic homeostasis and induce stress responses depending on the amplitude, frequency, and duration of the stress [17].

There have been studies comparing cold-hardened and nonhardened species between cultivars tolerant and non-tolerant to cold. Studies on cold-tolerant herbaceous plants after exposure to low temperatures have reported that reprogramming of carbon metabolism is involved in changes in gene expression, enzyme activity, and possibly changes of phosphate compartmentation [18]. Moreover, previous studies on winter rye leaves after growth at low temperatures have reported an increased in cell size, lower stomatal frequency, and an increase in chlorophyll content [19]. Plants can accumulate key metabolites [20] produced by enzymatic (e.g., CAT, POD, SOD) and non-enzymatic (e.g., ascorbic acid, carotenoids, tocopherols, and flavonoids) antioxidative components [21, 22] that work together to detoxify reactive oxygen species (ROS) [22] under biotic/abiotic stress conditions. Chlorophylls, carotenoids, and anthocyanins found in seed coats [23] are important antioxidants [24] known to be associated with seed germination behavior [25, 26] due to their antioxidant activities. Flavonoids is a group of natural substances consisting of variable phenolic structures and their subgroups, including flavones, flavanones, flavanonols, flavanols (catequins), anthocyanins, and chalcones [27]. They might be involved in the protective mechanism during freezing by scavenging ROS [28–31].

Transcriptions factors (TFs) can regulate gene expression by coordinating mechanisms involved in DNA methylation, chromatin organization, dimerization, and sequence-specific DNA binding. TFs can function as activators or repressors of genes [32]. The CBF/DREB1 family is the most studied transcription factor family responsible for cold hardening and frost tolerance. *WSC120* is similarly induced by cold and freezing temperatures as well as after cold [33–35]. Recent studies have reported that TFs involved in anthocyanin biosynthesis pathway are highly expressed in cold acclimated plants [36].

In this study, we hypothesized that the darker the seed coat color the better germination performance under low temperature conditions than those from other groups. Moreover, to study the acclimation in relationship to the different seed coat colors on the germination ability and seedling performances under the cold temperatures, two-group experimental designs were used to analyze how seed coat color and germination temperature could affect responses of seedlings to a chilling stress (CS). First, we performed inheritance analysis of deep purple seed coat color using F_3_ population obtained from the cross between yellow and deep purple seed inbreds. Second, segregating seeds were divided into three groups: yellow (Ye), medium purple (MP), and deep purple (DP). Seeds in each group were germinated under chilling-acclimated (CA) (4 °C) and non-acclimated (NA) conditions (18 °C) for a week, followed by normal growth for three weeks under control conditions. Finally, a second CS was induced for 6 h at 4 °C. This work aimed to evaluate the effects of chilling stress in different seed coat colors under non- acclimated and chilling-acclimated seeds and seedlings were evaluated in chlorophyll content, enzymatic activity, and gene expression of flavonoid related pathway, ROS scavenging enzyme genes and cold responsive genes in order to better characterize the seed coat color response to chilling stress and identify limiting factors useful for developing breeding strategies in order to improve the yield of wheat under chilling stress.

## Results

### Inheritance of purple seed color

Color segregation analysis was performed by bare eyes. Observers performed the analysis by dividing the whole set of seeds by plant into different color categories from 1 (yellow) to 9 (deep purple). After performing the analysis, the average score of three replicates was calculated and reported in Additional file 1: Table S1 and Additional file 2: Table S2.

**Fig. 1:**
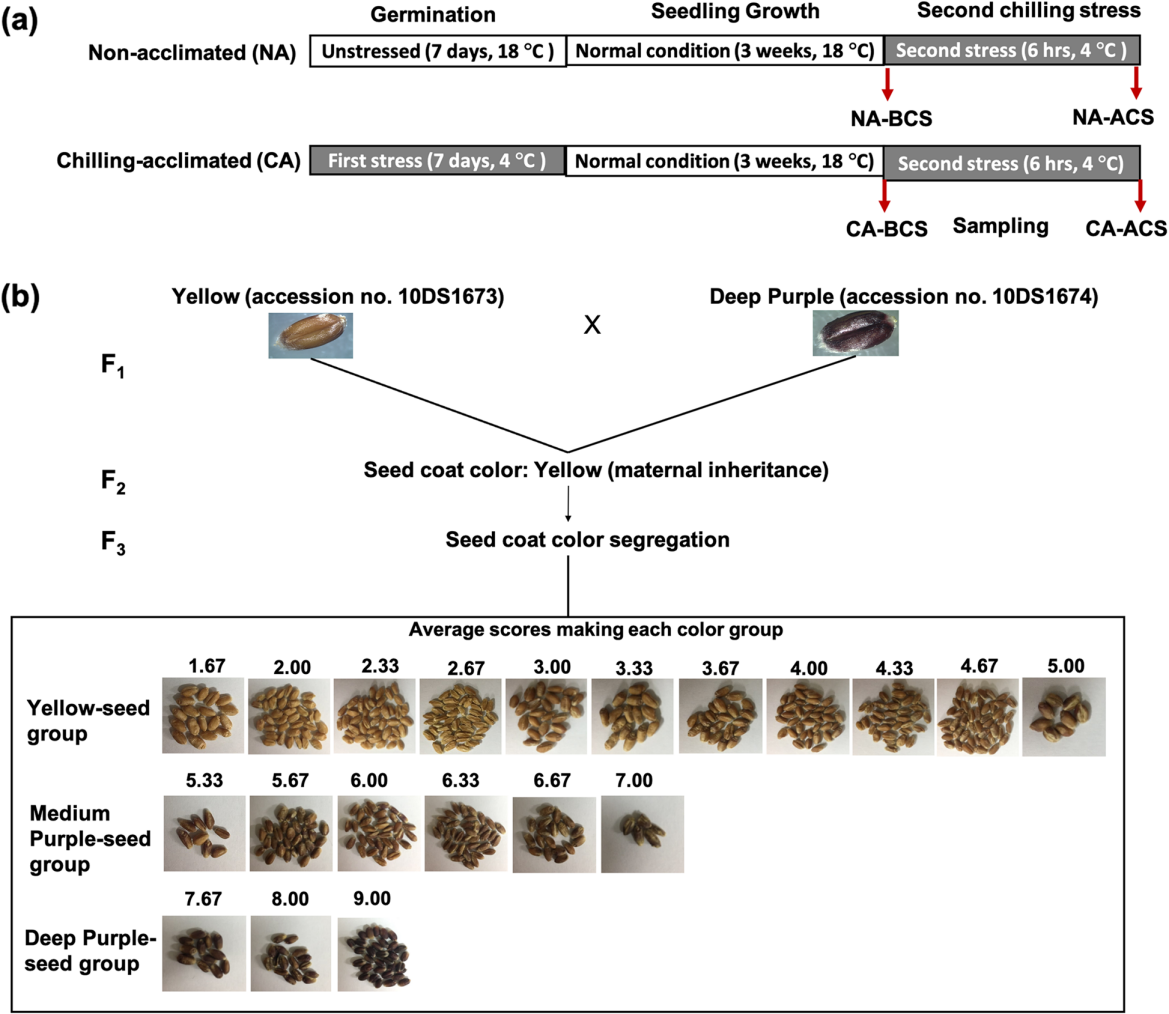
Experimental design and crosses of RILs for color segregation. **a** Schematic representation of experimental design and treatments. NA: non-acclimated, chilling acclimated (CA), BCS: before chilling stress, ACS: after chilling stress; (**b**) crosses for the generation of the seed coat color segregation and seed coat color segregation groups based on our genetic analysis. Seed coat color segregation, bulked groups from average scores making each groups: yellow seeds from scores from 1 to 5; medium purple seeds, scores > 5 to 7, and deep purple seeds, scores > 7 to 9

Results from the genetic analysis showed that the best fit for our seeds was the ratio of 13 Ye: 3 DP with a χ^*2*^ of 3.413 and a *p*-value of 0.06 (Table 1). However, we followed our second best χ^*2*^ result based on seed color variations observed in Fig. 1b, classifying our seeds as from the average scored Ye-seed (from 1–5), MP-seed (above 5 to 7), and DP-seed (above 7 to 9) with 12:3:1 ratio, χ^*2*^ of 1.988, and a *p* value of 0.37 (Table 1). We suggested the two-gene model as shown in Table 2 where dominant allele *A* resulted in Ye-seed coat color phenotype and genotypes *A_B* or *A_bb*, regardless of the genotype at a second locus *B*. In the absence of the dominant *A* allele (the *aa* genotype), *BB* or *Bb* resulted in MP-seed coat color phenotype (*aaB_*), whereas *bb* resulted in DP-seed color coat (*aabb*).

**Table 1:** Chi-square (χ^*2*^) analysis for the F_3_ generation for seed color segregation

Generation	Grain color				Total	Segregation ratio	χ^2^	Df	*P*-value
**F**_**1**_	Yellow X Deep Purple				-	-	-	-	-
**F**_**2**_	Yellow				-	-	-	-	-
**F**_**3**_		Yellow	Medium Purple	Deep Purple					
	Observed	111	-	30	141	13:3 ^a^	3.413	1	0.06
	Expected	114.5625	-	26.4375	141				
	Observed	111	25	5	141	12:3:1 ^b^	1.988	2	0.37
	Expected	105.75	26.4375	8.8125	141				

^a^ Seed scores were grouped in yellow from scores 1 to 5.0 and deep purple from > 5.0 to 9.0; ^b^ seed scores were grouped in yellow from scores 1 to 5.0, medium purple > 5.0 to 7.0 and deep purple from > 7.0 to 9.0

**Table 2:** Genotypic ratios of crosses illustrating the propose model for the inheritance in seed coat color

Category of cross	F_3_ ratio	Genotypes			Type of interaction
Dihybrid	13 Yellow	*A_B*	*A_bb*	*aaB_*	Dominant & recessive epistasis
	3 Deep Purple	*aabb*			
	12 Yellow	*A_B*	*A_bb*		Dominant epistasis
	3 Medium Purple	*aaB_*			
	1 Deep Purple	*aabb*			

### Phenolics and anthocyanins quantification

**Fig. 2:**
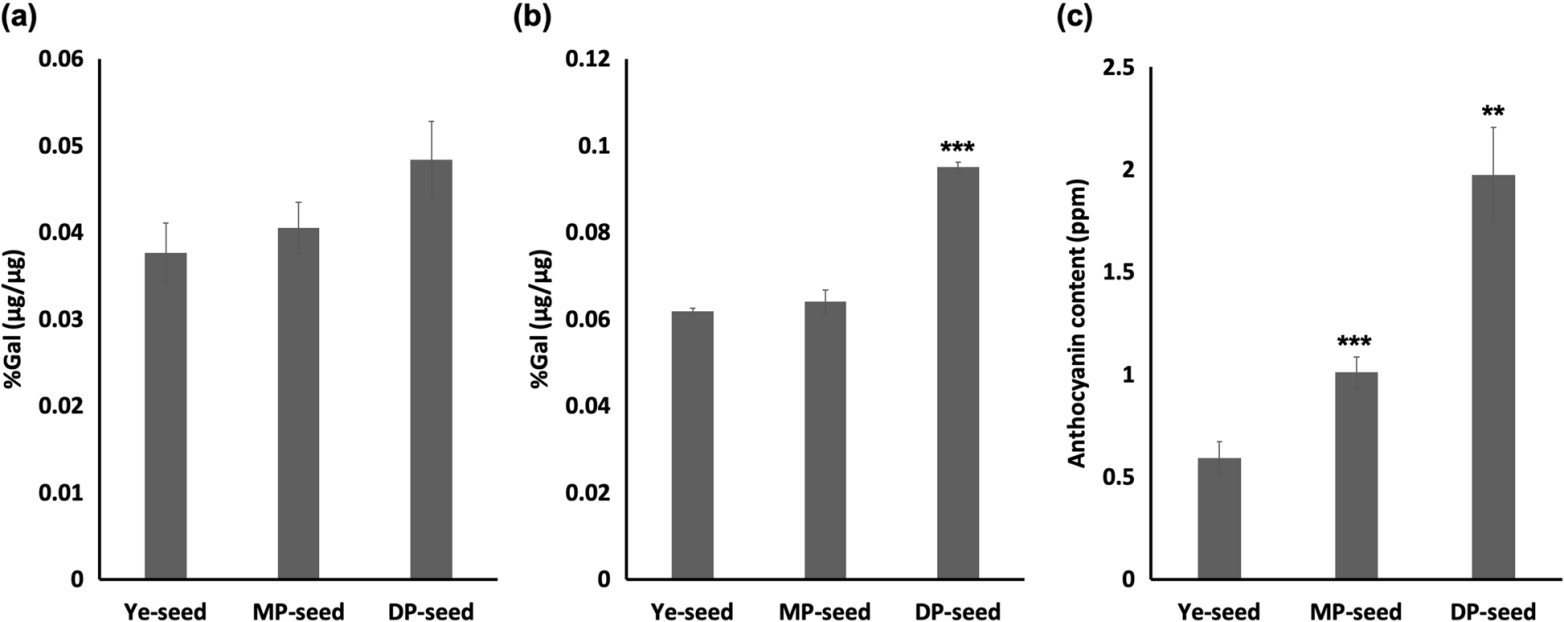
Phenolics and anthocyanins quantification in seeds. **a** Free phenolics, (**b**) total phenolics, and (**c**) anthocyanin quantification in yellow (Ye)-, medium purple (MP)- and deep purple (DP)- seed groups. Data are means ± SEM of three replicates. Significant differences, evaluated by t-test, are marked by asterisks: *p ≤ 0.05, **p ≤ 0.01, ***p ≤ 0.001 when compared to Ye-seed

We bulked our seeds based on the bared eyes scores and the genetic analysis to generate groups (Ye-, MP- and DP-seed) and extracted free phenolics (FP), total phenolics (TP), and total anthocyanin (AC). Although DP-seed group had the highest amount of FP, DP-FP showed no significant difference between MP- and Ye-seed groups. For TP, the DP-seed group also showed the highest content. However, it was significantly different from TP in other groups (*p* ≤ 0.05) (Fig. 2a and b). Regarding anthocyanin content, it was significantly higher among all groups (*p* ≤ 0.05) with 1.974, 1.010, and 0.593 ppm in DP-, MP-, and Ye-seed groups, respectively (Fig. 2c). These results showed that TP, and AC contents increased with deepening purple seed coat color.

### Germination rate of non- and chilling- acclimated conditions

**Fig. 3:**
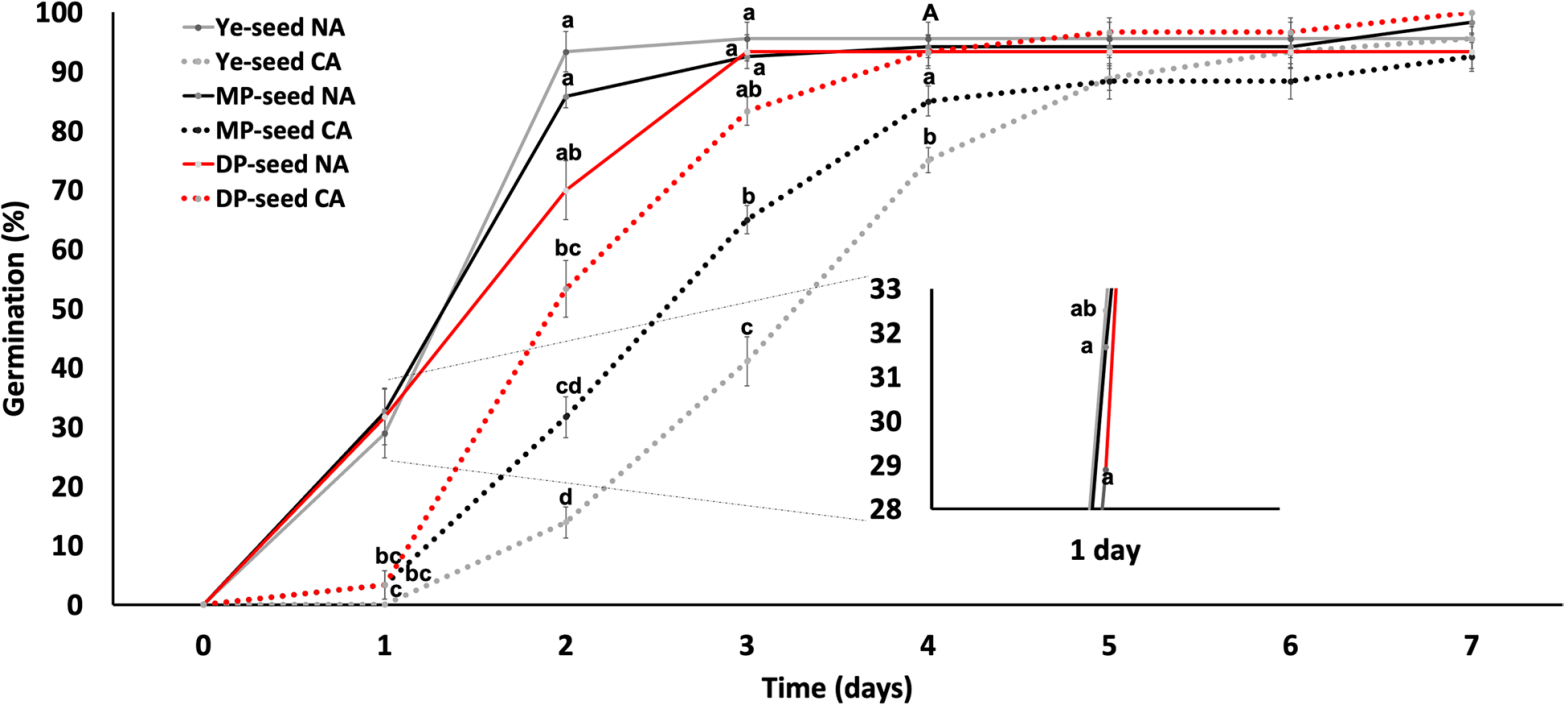
Germination percentage under non-acclimated and chilling acclimated seeds. Germination percentage per day in yellow (Ye-seed), medium purple (MP-seed), and deep purple seed groups (DP-seed) under non-acclimated (NA) and chilling-acclimated (CA) conditions. Data are presented as means ± SEM of three replicates. Different letters indicate significant differences in each coordinate value between groups (*p* ≤ 0.05).”A” means no significant difference between Ye-, MP-, and DP- seed under non-acclimated and DP-seed groups under CA conditions

Daily germination percentage was compared under NA and CA conditions among Ye-, MP-, and DP-seed groups. There were no visible differences in germination percentages on the first day (Additional file 3: Fig. S1). However, germination percentages started to show significant differences from the second day among the three groups. Daily germination percentage of the DP-seed group under CA condition was statistically similar to that of the DP-seed group under NA condition. Thus, germination performance of the DP-seed group was not affected by CS compared to other seed coat color groups. This was noted starting from day 2 when the DP-seed group under chilling germination already had germination rate of 53.33% compared to germination rates of 31.66% and 12.88% in MP- and Ye-seed groups, respectively. On day 3 under CA condition, DP-, MP-, and Ye-seed groups had germination rates of 83.33%, 65%, and 41.11%, respectively. This pattern was kept under the CA germination condition, with DP-seed germination percentage being higher compared than MP- and Ye-seed groups and the MP-seed group had higher germination rate until day 5 than the Ye-seed. However, on day 6, the MP-seed group did not show any more change in germination rate. Furthermore, after the fifth day, there was no significant difference in germination rate among Ye-, MP-, and DP-seed groups under NA or CA condition. Germination rates reached about 90% in all seed groups (Fig. 3).

### Chlorophyll contents in seedlings

Chlorophyll (*Chl*) has been used as an indicator for stress and chilling conditions of different crops [30, 37, 38]. We analyzed whether chlorophyll content was affected by seed coat color and acclimation by measuring chlorophyll contents in seedlings of seeds germinated under NA and CA conditions by measuring *Chla*, *Chlb*, *total Chl* and *Chla/Chlb* ratio.

Table 3 shows that there were no significant differences between the NA-BCS and CA-BCS seed seedlings between colors, moreover, there were also no significant differences after the chilling stress under NA- and CA- conditions between seed color groups.

**Table 3:** Chlorophyll content in seedlings after the second chilling stress

		**NA-Ye**	**CA-Ye**	**NA-MP**	**CA-MP**	**NA-DP**	**CA-DP**
**Chlorophyll a (μg ml-1FW)**	**BCS**	20.35 ± 6.52	19.27 ± 0.86	25.31 ± 8.78	15.53 ± 1.77	11.06 ± 0.71	11.47 ± 8.01
	**ACS**	18.85 ± 2.01	22.12 ± 2.74	17.76 ± 7.75	22.5 ± 4.63	26.97 ± 6.63	17.3 ± 5.27
**Chlorophyll b (μg ml-1FW)**	**BCS**	8.83 ± 3.27	6.03 ± 1.78	8.55 ± 3.61	6.82 ± 1.84	0.71 ± 0.99	1.71 ± 4.49
	**ACS**	6.68 ± 4.77	9.79 ± 2.17	8.53 ± 6.21	8.16 ± 0.85	4.3 ± 3.28	3.93 ± 1.25
**Total chlorophyll (μg ml-1FW)**	**BCS**	29.17 ± 9.79	25.3 ± 2.58	33.86 ± 12.36	22.35 ± 1.34	11.77 ± 0.31	13.18 ± 12.15
	**ACS**	25.53 ± 6.78	31.91 ± 4.42	26.29 ± 13.87	30.66 ± 4.52	31.26 ± 5.98	21.23 ± 6.00
**Chlorophyll a /chlorophyll b**	**BCS**	2.55 ± 0.27	4.01 ± 1.18	4.03 ± 1.39	2.64 ± 0.79	8.52 ± 7.65	1.93 ± 2.61
	**ACS**	5.8 ± 5.05	2.52 ± 0.48	3.54 ± 2.84	5.62 ± 5.85	3.93 ± 4.11	5.15 ± 1.58

Effect of the second chilling stress by measuring chlorophyll content in seedlings of yellow- (Ye-), medium purple- (MP-), and deep purple (DP-) seed seedlings. Chlorophyll a, chlorophyll b, total chlorophyll, and chlorophyll a/chlorophyll b ratio, samples were taken before chilling stress (BCS) (18 °C) and after chilling stress (ACS) (4 °C for 6 h) under non-acclimated (NA) (18 °C) and chilling-acclimated (CA) (4 °C) conditions. Data are means ± SD of three biological replicates. Significant differences, evaluated by t-test, are marked by asterisks: *p** ≤ **0.05, when compared to NA-BCS vs. CA-BCS and NA-ACS vs. CA-ACS within seed color group

### ROS scavenging enzymatic activity in seedlings

The activity of ROS scavenging enzyme of each seed coat color group was measured to analyze the relationship between seed coat color and antioxidant scavenging activity affected by CA during germination stage in seedlings. Table 4 shows total CAT, POD, and SOD levels in seedlings germinated under NA and CA conditions. CAT activity was highly increased in NA-ACS compared to CA-ACS in DP-seed seedlings ((*p* ≤ 0.01) from 0.53 U/gFW to 257.513 U/gFW), respectively. Whereas in MP-seed seedlings there was a significant increase from NA-ACS to CA-ACS (*p* ≤ 0.05), with 0.711 U/gFW to 222.428 U/gFW, respectively. Even though there was no significant differences in total POD between the groups, it can be noted that ACS, DP-seed seedlings had the highest total POD (7.638 U/gFW), compared to Ye- or MP-seed seedlings ACS. In case of total SOD BCS, MP-seed seedlings under NA conditions had the highest amount at 5.685 U/gFW, compared to Ye- and DP-seed seedlings. Moreover, ACS, DP-seed seedlings germinated under CA conditions had the highest total SOD ACS with 7.865 U/gFW, but these results are not statistically significant.

**Table 4:** Effect of second chilling stress in seedlings on ROS scavenging enzymes

		**NA-Ye**	**CA-Ye**	**NA-MP**	**CA-MP**	**NA-DP**	**CA-DP**
**Total CAT U/gFW**	**BCS**	0.242 ± 0.131	0.284 ± 0.217	0.702 ± 0.032	0.312 ± 0.127*	0.325 ± 0.0305	0.176 ± 0.04
	**ACS**	0.609 ± 0.276	0.684 ± 0.047	0.711 ± 0.109	222.428 ± 18.284**	0.53 ± 0.159	257.513 ± 20.042**
**Total POD U/gFW**	**BCS**	5.153 ± 0.876	6.275 ± 1.931	5.294 ± 0.529	6.907 ± 2.525	5.212 ± 2.235	7.102 ± 1.52
	**ACS**	4.59 ± 0.141	5.997 ± 2.403	4.631 ± 1.399	5.66 ± 0.468	7.023 ± 1.209	7.638 ± 1.13
**Total SOD U/gFW**	**BCS**	4.816 ± 1.276	3.303 ± 1.089	5.685 ± 0.500	6.18 ± 1.352	4.233 ± 1.054	4.49 ± 0.582
	**ACS**	6.19 ± 1.065	5.878 ± 1.08	4.855 ± 1.123	6.07 ± 1.968	5.618 ± 1.296	7.865 ± 1.213

Catalase (CAT), peroxidase (POD) and superoxide dismutase (SOD) quantification in yellow- (Ye-), medium purple- (MP-), and deep purple- (DP-) seed groups of seedlings germinated under non-acclimated (NA) (18 °C) and chilling acclimated (CA) (4 °C) conditions. Data are means ± SD of three biological replicates. Significant differences, evaluated by t-test, are marked by asterisks: *p ≤ 0.05 or **p ≤ 0.01 when compared when compared to NA-BCS vs. CA-BCS and NA-ACS vs. CA-ACS within seed color group

### Expression of ROS scavenging genes, transcripts related to flavonoid biosynthesis pathway, and cold response genes

**Fig. 4:**
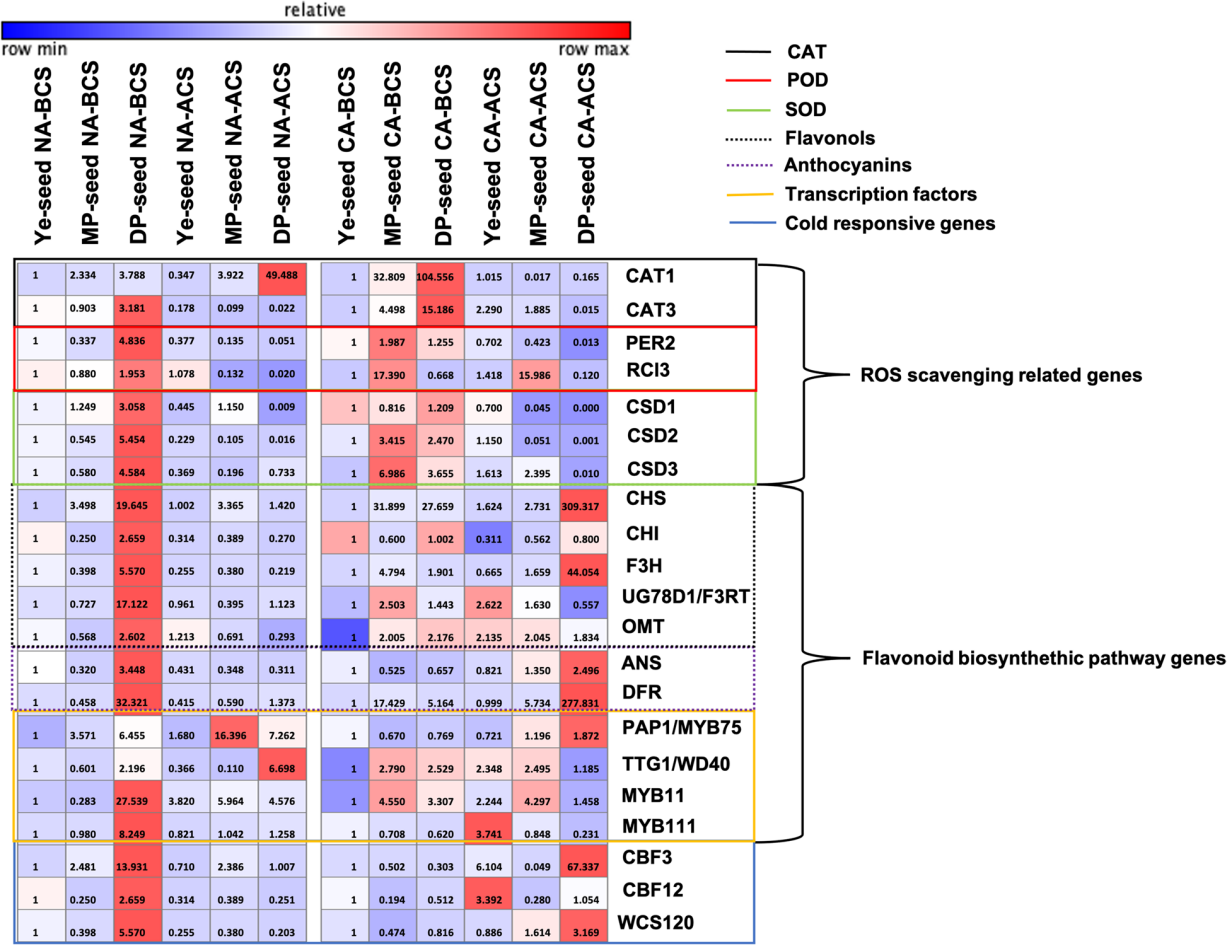
Heatmap showing the effect of the second chilling stress in gene expression. ROS (reactive oxygen species) scavenging genes; flavonoid, anthocyanins, and transcription factors regulating the flavonoid biosynthesis pathway; and cold response genes were measured in yellow- (Ye-), medium purple- (MP-), and deep purple- (DP-) seed groups of seedlings germinated under non-acclimated (NA) (18 °C) and chilling acclimated (CA) (4 °C) conditions before chilling stress (BCS) (18 °C) and after chilling stress (ACS) (4 °C for 6 h). Ye BCS and Ye ACS sample were used for comparison within groups (NA and CA). Numbers represent the fold changes. Relative color values are based of the subtraction of the row median, divided by row standard deviation. Data are presented as means ± SD of three biological replicates

The heatmap of gene expression patterns related in ROS scavengers, flavonoid biosynthesis, and cold responses are shown in Fig. 4. Under NA condition, the highest gene expressions were found mostly in the NA-BCS DP-seed seedlings, with exception for *CAT1*, *PAP1/MYB75*, and *TTG1/WD40*. The expression of flavonoid biosynthesis genes*,* such as *ANS, DFR, CHS*, *CHI* and *F3H,* increased in the DP-seed seedlings under CA condition after the second chilling stress, whereas expression of the ROS scavenging related genes were reduced in DP-seed seedlings of CA-ACS. In case of transcription factors involved in the flavonoid biosynthetic pathway, *PAP1/MYB75* showed highest expression in DP-seed seedlings of CA-ACS, however, expressions of *TTG1/WD40*, *MYB11*, and *MYB111* were decreased in DP-seed seedlings of CA-ACS. Among the cold responsive genes, *CBF3* and *WCS120* in DP-seed seedlings showed high expression in DP-seed seedlings of CA-ACS.

## Discussion

### Seed coat color scoring and genetic analysis

Seed color has been measured visually in several crops [39, 40]. For our experimental purposes, color was also evaluated by visual inspection, generating seeds scores from 1 to 9, where 1 represented yellow and 9 indicated deep purple of F_3_ seeds (Additional file 1: Table S1). Seed color is known to be determined by maternal genotype [41–43]. In the present study, F_3_ seeds (Fig. 1b) were analyzed for color segregation using Chi Square (χ^2^). χ^2^ results showed a 13: 3 (yellow: purple) ratio in this study (Table 1) that was previously reported in crosses between purple and blue wheat grains [42], with purple grains showing inhibiting effect on blue ones. Hence, in our materials, yellow grains had inhibiting effects on purple grains. It is well-known that this kind of ratio only two phenotypes segregate [44]. Therefore, we considered our second best χ^2^ result, identifying that the DP-seed color was controlled by a two gene interaction (12:3:1) from the cross between Ye-seed and DP-seed colored inbreds. F_2:3_ seeds were assigned into three groups based on seed coat color with a 12:3:1 ratio [yellow (Ye-seed), medium purple (MP-seed), deep purple (DP-seed), respectively] (Table 1), which has been demonstrated to be a characteristic of dominant epistasis usually having three phenotypes [44]. Our experiments and seed group color clusters were based on this segregation ratio. Therefore, we propose a dominant epistasis model of wheat seed color that Ye-seed color contained a dominant allele (A). In the presence of the dominant allele *A*, Ye-seed coat was produced as *A* allele was epistatic to both *B_* and *bb* combination. In the absence of the dominant *A* allele (the *aa* genotype), *BB* or *Bb* resulted in MP-seed color, while *bb* resulted in DP-seed color **(**Table 2**)**. This result is similar to a genetic model found for purple color deposition in rice pericarps [45].

### Phenolics and anthocyanins in seeds

Phenolics have been studied in yellow and black *Brassica napus* [46] as well as in colored maize [47]. Both studies have shown that darker colored seeds (black and purple) have higher phenolic contents. Our study showed similar results, with DP-seed group having higher FP and TP contents, followed by MP- and Ye-seed groups, but just TP results were statistically significant (Fig. 2a and b). Similarly, contents of anthocyanins were higher in deeper colored seeds, showing higher antioxidant activities than lighter ones [47–49]. These results are in accordance with our data, with DP-seed group had higher contents of anthocyanins, followed by MP- and Ye-seed groups (Fig. 2c).

### Germination

Under harsh conditions, it has been reported that seeds showing high antioxidant activity perform better due to scavenging of ROS [29, 36, 47]. We performed germination assay under chilling-acclimation (CA) (4 °C) and non-acclimated (NA) (18 °C) for a week. We hypothesized that darker colored seeds would show the fastest germination under low temperatures due to anthocyanins presented on their seed coats. As shown in Fig. 3, normal environmental conditions induced the germination of lighter-colored seeds (yellow seeds), whereas under chilling conditions, the germination ratio of darker-colored seeds (deep purple) was higher than that of yellow-colored seeds.

### Chlorophyll contents in seedlings after CS under NA and CA conditions

Chlorophyll (*Chl*) has been used as an indicator for stress and chilling conditions in different crops [30, 37, 38]. Our results showed that *Chl* contents in wheat leaves under BCS were different between germination under NA and CA conditions. Under NA conditions, contents of *Chla*, *Chlb*, *total Chl*, and *Chla*/*Chlb* ratio were higher (Table 3). On the other hand, when seeds were germinated under CA condition, results showed that *Chl* contents (*a*, *b*, *total*, and *a/b ratio*) decreased than those under NA condition in Ye- and MP-seed seedlings before CS. However, there was an increase of *Chl* contents in DP-seed seedlings. However, these results show no significant difference. We speculate these results are due to the short period of chilling temperature exposure.

### ROS scavenging enzymes

Stress induces the plant defense mechanisms and production of secondary metabolites [50]. ROS scavenging enzymes were quantified to verify effects of antioxidants on seedlings acclimation. Under the condition of ACS, the highest amount of ROS scavenging enzymes was detected in DP-seedling (CAT, POD, and SOD levels of 257.513, 7.638, and 7.865 U/gFW, respectively), higher than that under other conditions (Table 4). It has been described that the presence of ROS scavenging enzymes such as CAT, POD and SOD plays an important role in ROS detoxification as a defense mechanism against various stresses [51, 52]. However, these results were not in accordance with gene expression results by qRT-PCR when comparing NA-BCS and CA-BCS conditions. qRT-PCR results showed that under NA conditions BCS, with exception of *CAT1,* the rest of ROS related genes showed the highest expression in DP-seed seedlings (Fig. 4). In Table 4 it is shown that the CA-ACS in MP- and DP-seed seedlings that the CAT enzyme activity was dramatically increased compared to the Ye-seed seedlings and the NA condition. Prasad et al. [53] reported in acclimated and non-acclimated maize seedlings, that the CAT activity increased after treatment of maize seedlings for 7 days with temperature of 4 °C, concluding that for an unknown reason, there was no increase of *CAT3* expression in non-acclimated seedlings, suggesting that *CAT3* expression is post-transcriptionally or post-translationally controlled.

Based on these results, we can conclude that DP-seed seedlings have high ROS enzyme activities under normal conditions. A high ROS scavenging activity in DP-seed could maintain redox homeostasis to protect cell damage caused by various stresses.

Our results of NA and CA conditions after CS showed that exposure to low temperatures promoted the accumulation of POD and SOD related genes in MP- and DP-seed seedlings germinated under NA conditions. For POD related genes, *PER2* and *RCl3* were highly expressed in MP-seed seedlings. For SOD genes, *CSD2* and *CSD3* were also highly expressed in MP-seed seedlings*,* whereas *CSD1* was highly expressed in DP-seed seedlings. A previous study has shown that cold-primed plants possess more effective reactive oxygen scavenging systems than non-primed plants due to increased activities of SOD, APX and CAT, resulting in a better maintenance in homeostasis of ROS production [54]. Similarly, it has been reported that exposure to low temperatures can lead to accumulation of ROS [33]. Based on these results, we can assume that DP-seed seedlings under normal conditions already have a better maintenance of ROS. Therefore, under chilling conditions, DP-seed seedlings were not as affected as lighter colored seed seedlings under low temperatures stresses.

### Flavonoid biosynthetic pathway and cold responsive genes

According to previous reports [29, 36], anthocyanin biosynthetic genes are strongly down-regulated in more freezing-sensitive accessions but are more induced in more freezing-tolerant accessions. Our results (Fig. 4) showed that most of flavonoid biosynthesis gene transcripts were highly expressed in DP-seed seedlings under NA-BCS condition. Under CA-ACS condition, DP-seed seedlings showed the highest expression levels of flavonoid biosynthetic genes. Additionally, we found that *CHI*, *UG78D1/F3RT*, and *OMT* were induced by low temperatures, showing increases of their transcript levels in seedlings under NA-ACS.

The MBW, MYB-bHLH-WD40 complex, is associated with the regulation of anthocyanin and proanthocyanins biosynthesis. These transcription factors are key for activation of genes related to the flavonoid biosynthetic pathway [55, 56]. Previous reports have demonstrated that R2R3-MYB, PAP1/MYB75, and MYB111 can control anthocyanin-specific biosynthetic steps [55, 57]. The present study showed that there was an increase in transcript level of *MYB11* but not *MYB111*. *MYB11* is known to regulate early steps of the flavonoid pathway by activating promoters of early genes such as *CHS*, *CHI*, and *F3H* [55]. We can conclude that MYB111 in wheat might be a regulator of the anthocyanin pathway, whereas MYB11 can act as a regulator for *CHI* in NA-ACS in DP-seed seedlings. [36] have also shown that anthocyanin biosynthesis transcripts are induced by low temperatures. They are also induced in more acclimated plants. Likewise, our results showed high expression levels of anthocyanin related genes (*ANS* and *DFR*) after cold acclimation in DP-seed seedlings germinated under CA condition after CS. Moreover, it has been demonstrated that TTG1/WD40 can interact with PAP1/MYB75 for anthocyanin biosynthesis [57]. However, in our results under CA-ACS condition, *PAP1/MYB75*, but not *TTG1/WD40,* was highly expressed in DP-seed seedlings, similar to results reported by [36]. We can conclude that the anthocyanin biosynthesis pathway might not be regulated by a MBW complex in wheat seedlings. Probably PAP1/MYB75 is a key transcription factor of anthocyanin biosynthesis regulation in wheat seedlings. Our results indicate that there might be an upstream regulator unidentified in DP-seed seedlings for the anthocyanin pathway in response to CS.

A previous research [36] has shown that *TTG1/WD40* and *OMT* are highly expressed under warm conditions but with minor changes in cold conditions. Contrary to that, *OMT* was only highly expressed in DP-seed seedlings germinated under NA before CS, but was induced after CS. Therefore, *OMT* and *TTG1/WD40* might be affected by cold in wheat.

CBF and WSC120 are genes that encode transcriptional activators which are similarly induced by cold and freezing temperatures [33–35]. We analyzed *CBF3*, *CBF12,* and *WSC120* transcripts to observe low temperature responses of our NA and CA seedlings. Similarly, of cold responsive genes under BCS condition in NA plants, the highest transcripts of those genes were detected in DP-seed seedlings, whereas *CBF3* and *WCS120* showed high transcript levels in DP-seed seedlings germinated under CA condition in NA-ACS plants. *CBF12* was highly expressed in ACS condition of Ye-seed seedlings germinated under CA. Our results show that DP-seed seedlings NA-BCS had higher expression than CA-BCS, suggesting that the DP-seed seedlings might be in a state of innate pre-acquired stress response state under normal conditions. This data supports the results from DP-seed seedlings under CA-BCS where results showed that the gene expression was reduced in the cold responsive genes studied, showing that the chilling acclimation in DP-seed could endow the potence to meet with the stress resulting in reduce gene expressions. After the acclimation, another stress should enhance the cold genes expression response, which might result in a more efficient chilling stress response, as reported by [34] where the spring wheat cultivars have low tolerance to cold temperature compared to winter cultivars due to the inability to maintain low-temperature genes in an up-regulated state. Although *CBF12* and *WCS120* are known to be responsive to cold, the mode of expression might be different depending on the circumstances. As seen in Fig. 4, *CBF12* and *WCS120* gene expressions are higher in NA-BCS than CA-ACS for DP-seed seedlings, this may be due to the reasons stated above that the DP-seed seedlings are in a defensive state in NA-BCS. On the other hand, when comparing the gene expression level between CA-BCS and CA-ACS, there was an increase after the chilling stress, suggesting that the cold responsive genes in DP-seed seedlings were more responsive which could provide an effective tolerance ability of DP-seed. Also, based on these results, we suggest that *CBF3* could be the largely responsive gene in DP-seed seedlings in this study.

## Conclusions

Winter wheat is frequently exposed to a combination of low and subzero temperatures during early seedling stages. When the temperature is decreased gradually, wheat plants can acclimate to cold temperatures. However, sudden temperature changes can harm plants. Our results show that seed coat color has an impact on germination. Moreover, darker seed color seedlings can response better to lower temperatures. Seedlings from DP seeds under non-acclimated stated had increased of ROS scavenging enzymes, and transcription levels of genes involved in ROS scavenging, flavonoid biosynthetic pathway, and cold response suggesting that deep purple colored seed might be in a state of innate pre-acquired stress response state under normal conditions to counteract stresses in a more effective way. Whereas, after the acclimation, another stress should enhance the cold genes expression response, which might result in a more efficient chilling stress response in deep purple seed seedlings.

Low temperature conditions typically reduce physiological and biochemical processes. However, the present study showed that the darkest seed-color seedlings had better performances than lighter colored ones under chilling stress. Globally, low temperatures have a large impact on yields of crops. Thus, understanding seed coat colors and their benefits would be useful for generating crops with desired acclimation traits.

## Materials and methods

### Plant materials

Recombinant Inbred Lines (RILs) with different seed coat phenotypes, Yellow (accession no. 10DS1673, Korea University wheat sub-gene bank) and Deep Purple (accession no. 10DS1674), were used [58]. Seeds were germinated on moistened filter paper at room temperature for 24 h and vernalized at 4 °C in a dark chamber for 4 weeks. Each seedling was then transplanted to a pot (5 × 5 × 16 cm) filled with soil (Sunshine mix #1, Sun Gro Horticulture, Canada) in a well-controlled glasshouse at Korea University with a photoperiod of 16:8 h and temperatures between 20–25 °C. F_3_ seeds from 141 F_2_ plants were used for the analysis of seed coat color segregation. A detailed description of the material is shown in Additional file 1: Table S1.

### Genetic analysis of color segregation and germination assay

Color segregation analysis of the F_3_ population was performed by bare eye. F_3_ seeds from each 141 F_2_ plant were analyzed in nine different color categories (1: light yellow and 9: deep purple). Genetic analysis was performed using Chi Square (χ^*2*^) test in Microsoft Excel. Seeds were washed with commercial sodium hypochlorite (4%) for 3 min, rinsed with distilled water, and germinated in magenta boxes ﻿(6.5 × 6.5 × 20 cm; Greenpia Technology Inc., Yeoju, Korea) on a ﻿polypropylene mesh, keeping distilled water beneath the mesh. Non-acclimated (NA) conditions were performed in a chamber with a photoperiod of 16 h light: 8 h dark, temperature of 18 °C, humidity of 60%, and luminous power of 17,000 FLUX. Chilling-acclimation (CA) was performed in a fridge with a temperature of 4 °C for a week. Three replicates were performed for both NA and CA conditions.

Germination percentages [Germination Percentage = (Seeds germinated / Total seeds) * 100%] [59] were recorded daily for 7 days using radicle extrusion as a criterion [60, 61]. After 7 days of germination test, both NA and CA seedlings were transferred to a chamber with controlled photoperiod of 16 h light: 8 h dark, temperature of 18 °C, and humidity of 60% for three weeks. Both NA and CA groups were then exposed to a chilling stress (CS) for 6 h at 4 °C. Samplings for determining chlorophyll content, ROS, and RNA extraction for the gene expression were performed before and after CS as shown in Fig. 1a. Germination results are expressed as average germination of each color score bulked in yellow (Ye-seed, scores: 1–5), medium purple (MP-seed, scores: >5–7), and deep purple (DP-seed, scores: >7–9) groups based on genetic analysis.

### Quantification of phenolics and anthocyanins in seeds

Free phenolics (FP) and total phenolics (TP) were measured using the Folin-Ciocalteu reagent following the protocol of [62]. F_3_ bulked seeds from each group, Ye-, MP-, and DP-seed, were ground with liquid nitrogen and 20 mg grain powder was measured. Total anthocyanin content was determined according to the protocol of [63]. Absorbance was read at 765 nm for FP and TP whereas it was read at 530 nm and 657 nm for anthocyanins using a microplate reader (HIDEX-Sense 425–301, Finland). Results are expressed as the average of three biological replicates of phenolics or anthocyanin of each color score seeds bulked in Ye-, MP- or DP-seed groups based on genetic analysis.

### Chlorophyll content analysis for seedlings

Chlorophyll content was measured in triplicate using leave samples collected before and after the second CS from each color seed group, Ye-, MP-, and DP-seed. Samples were immediately frozen in liquid nitrogen and stored at -80 °C until further use. Chlorophyll content was performed using the method of [64] by measuring the absorbance at 652 and 665 nm with a microplate reader (HIDEX-Sense 425–301, Finland). Results are presented as the average of chlorophyll content of each color score seeds bulked in Ye-, MP- or DP-seed groups based on genetic analysis.

### ROS scavenging enzyme

Germination was performed in triplicate using RILs (accession no. 10DS1673 and 10DS1674), and the MP-seed group (F_3_ seeds generated from MP-seed scored seeds). Samples were taken before and after the second CS, frozen in liquid nitrogen, and stored at -80 °C until further use. Fresh tissue (0.5 g) was ground with liquid nitrogen and homogenized﻿ in 0.5 mL protein extraction buffer ﻿[0.2 M potassium phosphate buffer (pH 7.0) and 0.1 mM EDTA]. The Bradford method was used for total protein quantification followed by quantification of total superoxide dismutase (SOD), catalase (CAT), and peroxidase (POD) using the protocol of [58].

### RNA extraction, cDNA synthesis, and real-time PCR

Total RNA extraction was performed in triplicate from samples taken before and after the second CS from RILs (accession no. 10DS1673 and 10DS1674) and the MP-seed group (F_3_ seeds generated from MP scored seeds). Samples were ground in liquid nitrogen. TRIzol reagent (Invitrogen, USA) was used for RNA extraction. cDNA synthesis was performed using the cDNA Takara PrimeScript™ 1st strand cDNA Synthesis Kit (Takara, Japan). Gene-specific primers were designed using Primer-BLAST (NCBI, https://www.ncbi.nlm.nih. gov/tools /prime r-blast /) or from previous publications as described in Additional file 4: Table S3. Quantitative PCR was performed in triplicate using EvaGreen 2X qPCR MasterMix (ABM, Canada) on a CFX-96 RT-PCR machine (Bio-Rad, USA). *β-actin* (accession no*.* AB181991) was used as an internal control. Ct values for each gene were then normalized against actin expression. The 2^−ΔΔCT^ method was used to calculate expression levels in fold changes as previously described [65]. Heatmaps were created using Gene-E (https://software.broadinstitute.org/GENE-E/).

### Statistical analysis

All data are presented as means from three replicates. Significant differences were subjected to ANOVA, Duncan or t-test using R Studio version 1.2.5033 (http://www.rstudio.com/) or IBM® SPSS® Statistics for MAC version 25 (IBM Corp., Armonk, NY, USA). All tests were performed with 95%, 99% and 99.9% of confidence.

## Supplementary Information

Additional file 1: **Table S1.**  Description of the seed materials used for the study of the seed coat color segregation.

Additional file 2: **Table S2.** Summary of statistical analysis of seed coat color scoring by observers. 

Additional file 3: **Fig. S1.** Germination and recovery pictures. a) Germination under Non-Acclimated (NA) and Chilling-acclimated (CA) conditions; b) Images of the NA and CA seedlings after the 3 weeks of Normal condition before the chilling stress (BCS).

Additional file 4: **Table S3.**  List of primers used in this study for the qrt-PCR of the flavonoid related, ROS scavenging and cold responsive genes.

## Data Availability

All data generated during this study are included within the article and in its supplementary information files or are available from the corresponding author on reasonable request.

## Abbreviations

AC: Anthocyanin
ACS: After chilling stress
BCS: Before chilling stress
CA: Chilling-acclimated
Chl: Chlorophyll
CAT: Catalase
DP: Deep purple
FP: Free phenolics
MP: Medium purple
NA: Non-acclimated
POD: Peroxidase
ROS: Reactive oxygen species
SOD: Superoxide dismutase
TP: Total phenolics
Ye: Yellow
