# Supplementary material for: Effect of chilling acclimation on germination and seedlings response to cold in different seed coat colored wheat (Triticum aestivum L.)
Source: BMC Plant Biol. 2021 Jun 2;21:252. doi: 10.1186/s12870-021-03036-z (PMC8173842; doi:10.1186/s12870-021-03036-z)
Supplement: Supplementary file 1 — Table S1. Description of the seed materials used for the study of the seed coat color segregation. [file 12870_2021_3036_MOESM1_ESM.docx]

**Table S1.** Description of materials used in this study.

| **Pot no.** | **F_2_ seed coat color  (1: Yellow,  9: Deep Purple)** | **F_3_ seeds, seed coat color scoring (segregating population)** | | | |
| --- | --- | --- | --- | --- | --- |
|  |  | **Observer 1 seed coat color  (1: Yellow,  9: Deep Purple)** | **Observer 2 seed coat color  (1: Yellow,  9: Deep Purple)** | **Observer 3 seed coat color  (1: Yellow,  9: Deep Purple)** | **F_3_ seed coat color average score** |
| 1 | 6 | 3 | 2 | 2 | 2.33 |
| 2 | 6 | 3 | 2 | 2 | 2.33 |
| 3 | 6 | 3 | 2 | 3 | 2.67 |
| 4 | 6 | 3 | 2 | 2 | 2.33 |
| 5 | 6 | 3 | 1 | 1 | 1.67 |
| 6 | 6 | 4 | 5 | 4 | 4.33 |
| 7 | 6 | 5 | 5 | 5 | 5.00 |
| 8 | 6 | 3 | 3 | 2 | 2.67 |
| 9 | 6 | 6 | 6 | 6 | 6.00 |
| 10 | 6 | 7 | 7 | 7 | 7.00 |
| 11 | 6 | 5 | 5 | 6 | 5.33 |
| 12 | 6 | 3 | 1 | 1 | 1.67 |
| 13 | 6 | 5 | 4 | 4 | 4.33 |
| 14 | 6 | 4 | 3 | 3 | 3.33 |
| 15 | 6 | 7 | 5 | 7 | 6.33 |
| 16 | 6 | 7 | 6 | 6 | 6.33 |
| 17 | 6 | 4 | 2 | 3 | 3.00 |
| 18 | 6 | 5 | 4 | 4 | 4.33 |
| 19 | 6 | 3 | 2 | 3 | 2.67 |
| 20 | 6 | 7 | 5 | 6 | 6.00 |
| 21 | 6 | 3 | 2 | 2 | 2.33 |
| 22 | 6 | 3 | 2 | 2 | 2.33 |
| 23 | 6 | 8 | 7 | 7 | 7.33 |
| 24 | 6 | 3 | 2 | 3 | 2.67 |
| 25 | 6 | 4 | 2 | 3 | 3.00 |
| 26 | 6 | 7 | 6 | 7 | 6.67 |
| 27 | 6 | 5 | 4 | 5 | 4.67 |
| 28 | 6 | 4 | 3 | 4 | 3.67 |
| 29 | 6 | 3 | 1 | 2 | 2.00 |
| 30 | 6 | 3 | 2 | 2 | 2.33 |
| 31 | 6 | 3 | 2 | 2 | 2.33 |
| 32 | 6 | 7 | 7 | 8 | 7.33 |
| 33 | 6 | 6 | 4 | 5 | 5.00 |
| 34 | 6 | 7 | 6 | 6 | 6.33 |
| 35 | 6 | 4 | 3 | 2 | 3.00 |
| 36 | 6 | 3 | 2 | 2 | 2.33 |
| 37 | 6 | 8 | 8 | 8 | 8.00 |
| 38 | 6 | 4 | 2 | 3 | 3.00 |
| 39 | 6 | 6 | 5 | 6 | 5.67 |
| 40 | 6 | 2 | 2 | 2 | 2.00 |
| 41 | 6 | 3 | 3 | 3 | 3.00 |
| 42 | 6 | 3 | 3 | 3 | 3.00 |
| 43 | 6 | 3 | 2 | 3 | 2.67 |
| 44 | 6 | 3 | 3 | 3 | 3.00 |
| 45 | 6 | 6 | 6 | 6 | 6.00 |
| 46 | 6 | 5 | 5 | 5 | 5.00 |
| 47 | 6 | 3 | 2 | 3 | 2.67 |
| 48 | 6 | 3 | 3 | 3 | 3.00 |
| 49 | 6 | 5 | 5 | 5 | 5.00 |
| 50 | 6 | 4 | 2 | 3 | 3.00 |
| 51 | 6 | 3 | 3 | 3 | 3.00 |
| 52 | 6 | 2 | 2 | 3 | 2.33 |
| 53 | 6 | 4 | 2 | 4 | 3.33 |
| 54 | 6 | 5 | 4 | 5 | 4.67 |
| 55 | 6 | 4 | 4 | 3 | 3.67 |
| 56 | 6 | 3 | 3 | 2 | 2.67 |
| 57 | 6 | 7 | 6 | 6 | 6.33 |
| 58 | 6 | 6 | 6 | 6 | 6.00 |
| 59 | 6 | 3 | 2 | 3 | 2.67 |
| 60 | 6 | 2 | 1 | 3 | 2.00 |
| 61 | 6 | 2 | 2 | 2 | 2.00 |
| 62 | 6 | 3 | 3 | 3 | 3.00 |
| 63 | 6 | 8 | 8 | 8 | 8.00 |
| 64 | 6 | 4 | 2 | 3 | 3.00 |
| 65 | 6 | 5 | 4 | 5 | 4.67 |
| 66 | 6 | 6 | 4 | 5 | 5.00 |
| 67 | 6 | 7 | 6 | 6 | 6.33 |
| 68 | 6 | 2 | 2 | 2 | 2.00 |
| 69 | 6 | 2 | 2 | 2 | 2.00 |
| 70 | 6 | 3 | 3 | 3 | 3.00 |
| 71 | 6 | 2 | 2 | 2 | 2.00 |
| 72 | 6 | 3 | 2 | 3 | 2.67 |
| 73 | 6 | 7 | 5 | 6 | 6.00 |
| 74 | 6 | 3 | 2 | 3 | 2.67 |
| 75 | 6 | 6 | 5 | 6 | 5.67 |
| 76 | 6 | 2 | 1 | 3 | 2.00 |
| 77 | 6 | 4 | 2 | 3 | 3.00 |
| 78 | 6 | 4 | 3 | 3 | 3.33 |
| 79 | 6 | 4 | 3 | 3 | 3.33 |
| 80 | 6 | 5 | 4 | 4 | 4.33 |
| 81 | 6 | 2 | 3 | 3 | 2.67 |
| 82 | 6 | 3 | 3 | 3 | 3.00 |
| 83 | 6 | 7 | 6 | 6 | 6.33 |
| 84 | 6 | 4 | 3 | 3 | 3.33 |
| 85 | 6 | 4 | 3 | 3 | 3.33 |
| 86 | 6 | 2 | 2 | 2 | 2.00 |
| 87 | 6 | 6 | 6 | 6 | 6.00 |
| 88 | 6 | 3 | 2 | 2 | 2.33 |
| 89 | 6 | 2 | 3 | 3 | 2.67 |
| 90 | 6 | 4 | 2 | 3 | 3.00 |
| 91 | 6 | 3 | 2 | 3 | 2.67 |
| 92 | 6 | 2 | 2 | 2 | 2.00 |
| 93 | 6 | 4 | 3 | 4 | 3.67 |
| 94 | 6 | 5 | 4 | 5 | 4.67 |
| 95 | 6 | 4 | 3 | 3 | 3.33 |
| 96 | 6 | 3 | 2 | 1 | 2.00 |
| 97 | 6 | 3 | 3 | 3 | 3.00 |
| 98 | 6 | 6 | 5 | 5 | 5.33 |
| 99 | 6 | 4 | 3 | 3 | 3.33 |
| 100 | 6 | 2 | 3 | 2 | 2.33 |
| 101 | 6 | 2 | 2 | 2 | 2.00 |
| 102 | 6 | 5 | 4 | 5 | 4.67 |
| 103 | 6 | 3 | 1 | 3 | 2.33 |
| 104 | 6 | 5 | 4 | 4 | 4.33 |
| 105 | 6 | 5 | 4 | 4 | 4.33 |
| 106 | 6 | 3 | 3 | 3 | 3.00 |
| 107 | 6 | 4 | 3 | 3 | 3.33 |
| 108 | 6 | 6 | 5 | 5 | 5.33 |
| 109 | 6 | 3 | 2 | 3 | 2.67 |
| 110 | 6 | 3 | 3 | 3 | 3.00 |
| 111 | 6 | 4 | 3 | 3 | 3.33 |
| 112 | 6 | 3 | 2 | 3 | 2.67 |
| 113 | 6 | 4 | 4 | 4 | 4.00 |
| 114 | 6 | 8 | 7 | 8 | 7.67 |
| 115 | 6 | 5 | 4 | 5 | 4.67 |
| 116 | 6 | 4 | 3 | 4 | 3.67 |
| 117 | 6 | 5 | 3 | 4 | 4.00 |
| 118 | 6 | 2 | 2 | 2 | 2.00 |
| 119 | 6 | 2 | 2 | 2 | 2.00 |
| 120 | 6 | 6 | 5 | 6 | 5.67 |
| 121 | 6 | 3 | 3 | 3 | 3.00 |
| 122 | 6 | 7 | 5 | 6 | 6.00 |
| 123 | 6 | 5 | 4 | 4 | 4.33 |
| 124 | 6 | 3 | 2 | 2 | 2.33 |
| 125 | 6 | 3 | 2 | 3 | 2.67 |
| 126 | 6 | 5 | 4 | 4 | 4.33 |
| 127 | 6 | 2 | 3 | 3 | 2.67 |
| 128 | 6 | 5 | 4 | 5 | 4.67 |
| 129 | 6 | 4 | 5 | 4 | 4.33 |
| 130 | 6 | 4 | 4 | 4 | 4.00 |
| 131 | 6 | 6 | 6 | 6 | 6.00 |
| 132 | 6 | 6 | 5 | 5 | 5.33 |
| 133 | 6 | 3 | 3 | 3 | 3.00 |
| 134 | 6 | 4 | 3 | 4 | 3.67 |
| 135 | 6 | 3 | 3 | 3 | 3.00 |
| 136 | 6 | 3 | 4 | 3 | 3.33 |
| 137 | 6 | 2 | 2 | 3 | 2.33 |
| 138 | 6 | 3 | 3 | 3 | 3.00 |
| 139 | 6 | 6 | 5 | 5 | 5.33 |
| 140 | 6 | 7 | 7 | 6 | 6.67 |
| 141 | 6 | 3 | 1 | 3 | 2.33 |
