# Supplementary material for: Effect of chilling acclimation on germination and seedlings response to cold in different seed coat colored wheat (Triticum aestivum L.)
Source: BMC Plant Biol. 2021 Jun 2;21:252. doi: 10.1186/s12870-021-03036-z (PMC8173842; doi:10.1186/s12870-021-03036-z)
Supplement: Supplementary file 2 — Table S2. Summary of statistical analysis of seed coat color scoring by observers. [file 12870_2021_3036_MOESM2_ESM.docx]

**Table S2.** Summary of Statistical Analysis of Seed Coat Color Scoring by Observers.

| **Statistics** | | | | |
| --- | --- | --- | --- | --- |
|  |  | **Observer 1** | **Observer 2** | **Observer 3** |
| N | Valid | 141 | 141 | 141 |
|  | Missing | 0 | 0 | 0 |
| Mean | | 4.1418 | 3.4255 | 3.766 |
| Std. Error of Mean | | 0.13712 | 0.13551 | 0.13528 |
| Median | | 4 | 3 | 3 |
| Mode | | 3 | 2 | 3 |
| Std. Deviation | | 1.62824 | 1.60905 | 1.60641 |
| Variance | | 2.651 | 2.589 | 2.581 |
| Range | | 6 | 7 | 7 |
| Minimum | | 2 | 1 | 1 |
| Maximum | | 8 | 8 | 8 |
| Sum | | 584 | 483 | 531 |
| Percentiles | 25 | 3 | 2 | 3 |
|  | 50 | 4 | 3 | 3 |
|  | 75 | 5 | 4.5 | 5 |
