# Supplementary figures and images for: Effect of chilling acclimation on germination and seedlings response to cold in different seed coat colored wheat (Triticum aestivum L.)
Source: BMC Plant Biol. 2021 Jun 2;21:252. doi: 10.1186/s12870-021-03036-z (PMC8173842; doi:10.1186/s12870-021-03036-z)

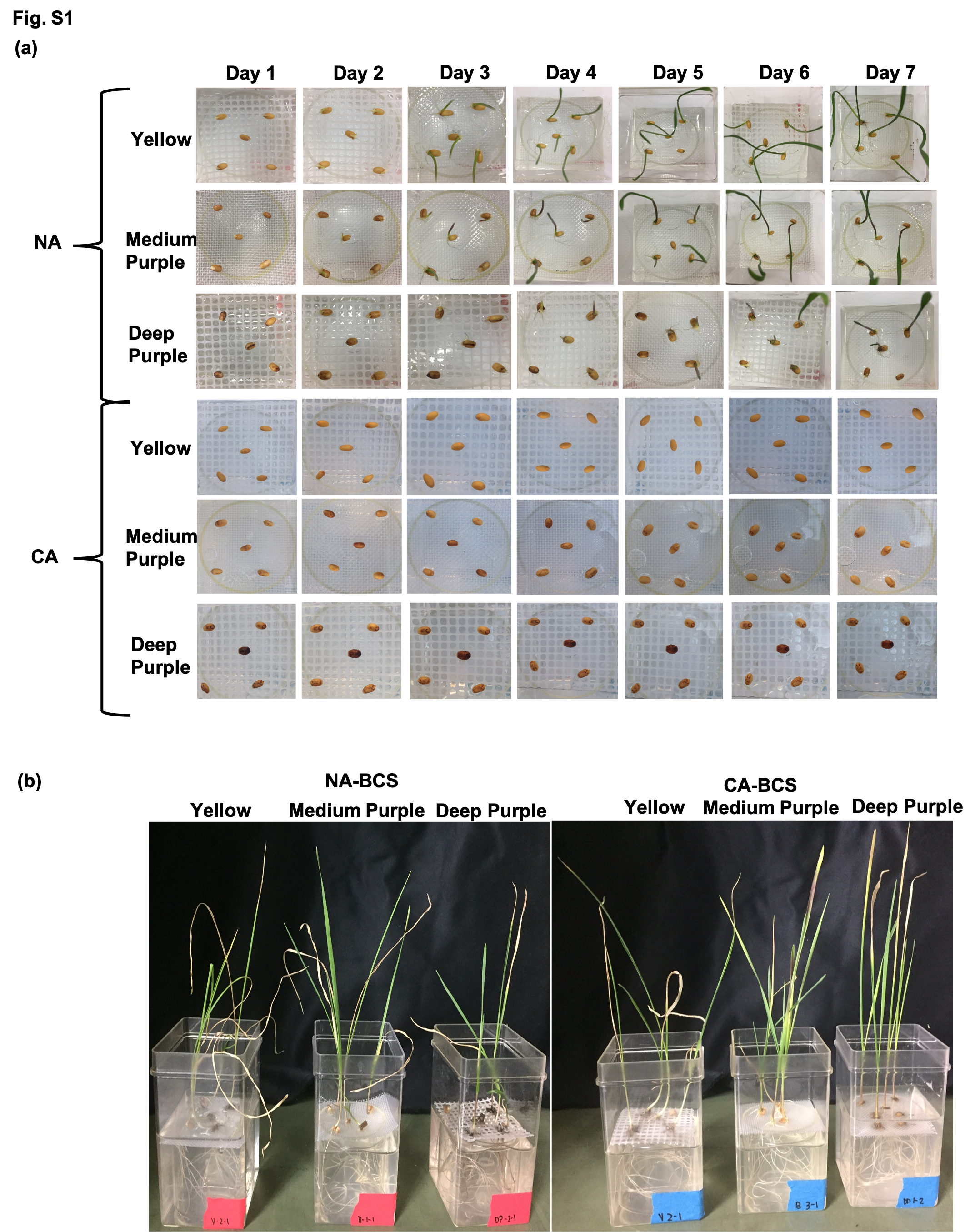

Supplement: Supplementary file 3 — Fig. S1. Germination and recovery pictures. a) Germination under Non-Acclimated (NA) and Chilling-acclimated (CA) conditions; b) Images of the NA and CA seedlings after the 3 weeks of Normal condition before the chilling stress (BCS). [file 12870_2021_3036_MOESM3_ESM.png]
