# Supplementary material for: Effect of chilling acclimation on germination and seedlings response to cold in different seed coat colored wheat (Triticum aestivum L.)
Source: BMC Plant Biol. 2021 Jun 2;21:252. doi: 10.1186/s12870-021-03036-z (PMC8173842; doi:10.1186/s12870-021-03036-z)
Supplement: Supplementary file 4 — Table S3. List of primers used in this study for the qrt-PCR of the flavonoid related, ROS scavenging and cold responsive genes. [file 12870_2021_3036_MOESM4_ESM.docx]

**Table S3.** List of primers used in this study.

|  | **Gene name** | **Primer sequence** | | **Reference** |
| --- | --- | --- | --- | --- |
|  |  | **F** | **R** |  |
| **Housekeeping gene** | *𝛃 -actin* | ACCCTCAGAGGAATAAGGGGT | ATACAGCAGGCAAGCACCAT | AB181991 |
| **qRT-flavonoid related** | *PAP1/MYB75* | TGTAAGAGCTGGGCTAAACC | GAAGATCGACTTCATCAGAGC | (Skirycz et al., 2007) |
|  | *TTG1/WD40* | TCGATATTCGTTCGCCGACT | GCCTGTGTATCATCACCACCAG | (Rubin et al., 2009) |
|  | *MYB11* | GGCGATTGTAACCCAAGCATT | TCACATGAGGACACGTGGACA | (Rubin et al., 2009) |
|  | *MYB111* | CGGCCTCACAATGTTTCTCAC | CCACTCATAAGGCCCCAAAGA | (Rubin et al., 2009) |
|  | *UGT78D1/F3RT* | TGCTGAAACACGAGGCAATG | ATCCGCCAAAATCGGTCTG | (Rubin et al., 2009) |
|  | *OMT* | CAATCCCGGAGGCAAAGAA | GAAGCCTGATGCTTTGGCTA | (Schulz et al., 2015) |
|  | *CHS* | ACTGGCGAGGGAATGGATTG | GTGACTGGGACGCTATGGAG | (Hong et al., 2018) |
|  | *CHI* | GCGTCGCCTACTGGAAG | GAAGCTGTGGGGTCCGAA | (Hong et al., 2018) |
|  | *F3H* | GGCCACGTCAGTACTCTCTTC | CTGCATGCACAAAGCACAGT | (Hong et al., 2018) |
|  | *DFR* | CACGAGGTACTTGACCGCAT | AGCTCCAGCAATGGCTTGTT | (Hong et al., 2018) |
|  | *ANS* | GTCCTCTCATCTGGTTTCGGG | GGTCACCCAGAAGGAGCCTA | (Hong et al., 2018) |
|  | *ANR* | ATACCTTCCCTCACTGCTGG | CCTTTCATTAGGGACGCAGC | (Hong et al., 2018) |
| **qRT-ROS** | *CAT1* | CGCCATGCCGAAAAATACCC | GCCTGTCTGAATCCCAGGAC | At1g20630 |
|  | *CAT3* | CTATCGGAGCCACGTCTCAC | CGGCTTGCAAGTTTCTGTCC | At1g20620 |
|  | *CSD1* | ACAGCAGTGAGGGTGTTACG | GGTGTCACCAAGAGCATGGA | At1g08830 |
|  | *CSD2* | TGGCTGCCACCAACACAA | CGGCGAAGGAAACAGATTGG | At2g28190 |
|  | *CSD3* | CGTCCGAGGCTGTCTTCAAT | AGCCAGGAGAGAGTCCTGAG | At5g18100 |
|  | *PER2* | GTGGCTCAGAAGAAAGGGCT | AGGGAGAATGGCCTGAGTCT | At1g05240 |
|  | *RCI3* | ACTATCCGGGGCTCACACTA | TGAGATTGGCTGCGTACTCG | At1g05260 |
| **qRT-cold responsive** | *CBF3* | AATCGTCGTCTGAGTCTGACAGTG | TTCCGGGAACAAGTCAAGCCT | (Sutton et al., 2009) |
|  | *CBF12* | AAATGGACGCGGGCACGTACTA | TCATCAGTGGTTCCATAGCGCC | (Sutton et al., 2009) |
|  | *WCS120* | AAGGATAAGCTGCCTGGACA | TCTCCTCCAACGACCAAGTG | M93342.2 |

Hong MJ, Kim DY, Ahn JW, Kang SY, Seo YW, Kim JB. Comparison of radiosensitivity response to acute and chronic gamma irradiation in colored wheat. Genet Mol Biol. 2018;41(3):611-623. doi:10.1590/1678-4685-GMB-2017-0189

Rubin G, Tohge T, Matsuda F, Saito K, Scheible WR. Members of the LBD family of transcription factors repress anthocyanin synthesis and affect additional nitrogen responses in Arabidopsis. Plant Cell. 2009;21(11):3567-3584. doi:10.1105/tpc.109.067041

Schulz E, Tohge T, Zuther E, Fernie AR, Hincha DK. Natural variation in flavonol and anthocyanin metabolism during cold acclimation in Arabidopsis thaliana accessions. Plant Cell Environ. 2015;38(8):1658-1672. doi:10.1111/pce.12518

Skirycz A, Jozefczuk S, Stobiecki M, et al. Transcription factor AtDOF4;2 affects phenylpropanoid metabolism in Arabidopsis thaliana. New Phytol. 2007;175(3):425-438. doi:10.1111/j.1469-8137.2007.02129.x

Sutton F, Chen DG, Ge X, Kenefick D. Cbf genes of the Fr-A2 allele are differentially regulated between long-term cold acclimated crown tissue of freeze-resistant and - susceptible, winter wheat mutant lines. BMC Plant Biol. 2009;9:34. doi:10.1186/1471-2229-9-34
